# Plasma BDNF and TrkB mRNA in PBMCs Are Correlated With Anti-depressive Effects of 12-Weeks Supervised Exercise During Protracted Methamphetamine Abstinence

**DOI:** 10.3389/fnmol.2020.00020

**Published:** 2020-03-06

**Authors:** Jue Yang, Jun Tan, Lan Zheng, Chun Xia Lu, Wen Qi Hou, Yi Liu, Qiu Fang Li, Jin Xiu Li, Dan Cheng, Xu Luo, Jun Zhang

**Affiliations:** ^1^Key Laboratory of Physical Fitness and Exercise Rehabilitation of Hunan Province, Hunan Normal University, Changsha, China; ^2^Franklin College of Arts and Sciences, University of Georgia, Athens, GA, United States; ^3^Hunan Judicial Police Vocational College, Changsha, China

**Keywords:** methamphetamine withdrawal, neurotrophin, neurotrophin receptor, exercise intervention, depression

## Abstract

**Purpose**: The aim of this study was to evaluate the potential neurotrophic factors and expression of neurotrophin receptors in peripheral blood mononuclear cells linked with the antidepressant action of exercise intervention during protracted methamphetamine (METH) abstinence.

**Materials and Methods**: A total of 72 male METH addicts, including 47 individuals with depression and 25 individuals without depression, were recruited in this study. Individuals with depression were divided into the depression control group and the depression exercised group. Consequently, 12 weeks of supervised exercise intervention was applied. Depression and anxiety were analyzed; plasma brain-derived neurotrophic factor (BDNF), neuronal growth factor (NGF), neurotrophin-3 (NT-3), NT-4, and proBDNF levels were tested using enzyme-linked immunosorbent assay; the mRNA expressions of TrkA, TrkB-FL, TrkB-T1, TrkCB, and P75NTR in peripheral blood mononuclear cells were detected by quantitative real-time polymerase chain reaction (qRT-PCR).

**Results**: NT-4 plasma levels were correlated with depression (*r* = −0.330, *p* = 0.005), which remained significant after Bonferroni correction. In addition, the BDNF and NT-3 levels in the plasma were significantly correlated with depression (*r* = −0.268, *p* = 0.023; *r* = −0.259, *p* = 0.028), but did not reach significance after Bonferroni correction. The BDNF, NT-3, and NT-4 plasma levels were significantly different between the depressive control group and the depressive exercise group using pre-exercise values as the covariate. The fold changes in TrkB-FL and TrkB-T1 mRNA in peripheral blood mononuclear cells between the post-exercise and pre-exercise demonstrated a remarkable decrease (fold change = −11.056 and −39.055).

**Conclusions**: Exercise intervention can alleviate depression and anxiety during protracted METH abstinence. Decrease in BDNF and the expression of TrkB in peripheral blood mononuclear cells occur following the exercise intervention.

## Introduction

Depression and anxiety are the most prominent psychiatric complaints reported by methamphetamine (METH) users (Glasner-Edwards and Mooney, 2014; Bagheri et al., 2015). Previous studies have shown a positive correlation between METH-withdrawal signs such as anxiety, depression, and obsessive-compulsive disorder with relapse and drug craving (Nakama et al., 2008; Shen et al., 2012). Thus, the treatment for anxiety and depression is important in preventing the METH relapse and for creating better outcomes during METH abstinence.

A growing evidence has suggested that neurotrophin, and especially brain-derived neurotrophic factor (BDNF), is important in anxiety and depression. The neurotrophin family includes four different members, including neuronal growth factor (NGF) (Cohen et al., 1954), BDNF (Barde et al., 1982), neurotrophin-3 (NT-3) (Maisonpierre et al., 1990), and neurotrophin-4/5 (NT-4/5) (Berkemeier et al., 1991; Ip et al., 1992). The neurotrophin actions are mediated by interaction with two transmembrane receptors. All members of the neurotrophin family bind to pan-neurotrophin receptor p75NTR with low affinity (Johnson et al., 1986; Radeke et al., 1987). However, only mature neurotrophins, which bind to different tropomyosin-related kinase (Trk) receptors with high affinity, exhibit ligand selectivity. TrkA has been reported as the preferred receptor for NGF (Kaplan et al., 1991; Klein et al., 1991), while trkB and TrkC have been identified to exert the effects of both BDNF and NT-4 (Ip et al., 1992; Klein et al., 1992), and NT-3 (Lamballe et al., 1991), respectively. Besides, pro-neurotrophins (immature form of neurotrophin), such as proBDNF, proNGF, and proNT-3, have been found to function via interaction with p75NTR (Fayard et al., 2005; Feng et al., 2010; Shen et al., 2013).

Previous studies on antidepressants for treatment of METH withdrawal, dependence, and co-occurring mood disorders have suggested that the existing behavioral and pharmacological treatments are ineffective (Hellem et al., 2015). Accumulating evidence has shown that BDNF is implicated in the pathophysiology of depression and the antidepressant effects of exercise (Heyman et al., 2012; Archer et al., 2014; Lu et al., 2014). In addition, previous studies have suggested that neurotrophins such as NGF, NT-3, and NT-4/5 are important factors for neuroplasticity regulation, which have an important role in the neurotrophic hypothesis of antidepression (Pae et al., 2008; Overstreet et al., 2010; Hochstrasser et al., 2013; Lang and Borgwardt, 2013; Liu et al., 2017). The aim of this study was to examine the association between the neurotrophins and the antidepressant action of exercise intervention during METH abstinence. First, we examined whether plasma neurotrophins (NFs) levels in withdrawal METH addicts are different in individuals with and without psychiatric symptoms (depression and anxiety) during long-lasting abstinence. Second, we investigated the effects of exercise training on plasma neurotrophins levels and degree of depression and anxiety. In addition, we explored the association between neurotrophins plasma levels and protracted withdrawal depression and anxiety after exercise intervention.

There is evidence that peripheral neurotrophins concentrations are associated with brain tissue expression. Previous studies have suggested that peripheral blood BDNF levels are correlated with brain tissue BDNF levels in rodents (Sartorius et al., 2009). Moreover, NGF, NT-3, and NT-4/5 can cross the blood–brain barrier of mice, thus reaching the brain parenchyma; while peripheral blood administration of neurotrophins could have neurobiological effects in the central nervous system (Pan et al., 1998). Based on the view that mood states are influenced by the communication between the immune system and the brain (Glaser and Kiecolt-Glaser, 2005; Ziemssen and Kern, 2007), the expression of neurotrophin receptors on immune cells that control a wide range of immune responses may mediate the interaction between the neuroregulation and the immunologic function. Therefore, in this study, we also investigated the neurotrophin receptor expression in peripheral blood mononuclear cells obtained from exercised abstinent METH individuals before and after exercise intervention. Besides, we examined the correlation between the expression of neurotrophin receptors in peripheral blood mononuclear cells and the protracted withdrawal of psychiatric symptoms. We believe that further understanding of relationships among neurotrophins plasma levels, expression of neurotrophin receptors in peripheral blood mononuclear cells, and protracted withdrawal symptoms including depression and anxiety during long-term recovery could be used to improve the recovery strategies.

## Materials and Methods

### Subjects

Forty-seven male METH-dependent individuals with depression (the score of SDS ≥50, age range 23–39 years, mean age 31.521 years) and 25 gender-matched METH-dependent controls without depression (the score of SDS <50, age range 25–38 years, mean age 31.320 years) from the Detoxification Rehabilitation Center of Baini Lake in Hunan province were enrolled in this study. METH-dependent individuals were isolated in the Detoxification Rehabilitation Center for 2 years, and no drugs were prescribed to these patients. Depression symptoms were measured by Zung Self-rating Depression Scale (Z-SDS), which is a self-reported 20-item questionnaire (Zung, 1965) containing item responses rated from 1 to 4. The overall score represents the severity of depressive symptoms, where a cutoff score of 40 indicates “clinically significant depression” (Zung, 1973). The index score of the scale is obtained when 1.25 multiplies the raw score. A total index score of 50 or higher was used as a cutoff point for depressive symptoms among the Chinese population. The Zung Self-rating Anxiety Scale (Z-SAS) was used to measure the anxiety symptoms; the Z-SAS questionnaire consists of 20 items scored from 1 to 4. The score from each item was calculated to obtain an overall score, and a higher overall score indicates higher levels of anxiety (Zung, 1971). Both Z-SDS and Z-SAS have been validated and extensively used in China (Chen et al., 2014; Liu et al., 2014).

All psychiatric interviews were conducted by trained psychiatrists using the Chinese version of the Structured Clinical Interview for Diagnostic and Statistical Manual (DSM-IV) Axis I disorders (SCID-I) for the diagnosis of mental disorders and schizophrenia. The Chinese version of SCID-I has been used in many epidemiological studies in China with excellent reliability and validity (Phillips et al., 2009). Exclusion criteria were as follows: (1) serious mental illnesses (schizophrenia, manic episodes, intellectual disability); (2) clinically significant cardiac or pulmonary disease screening based on clinical medical records; (3) musculoskeletal disease screened by checking medical records that would prevent participation in an exercise regimen; and (4) other medical conditions, ECG findings, or clinical laboratory results that would compromise the safety of a study participant.

This study was conducted in compliance with the Helsinki Declaration. The Institutional Review Board at the Hunan Normal University approved the study protocol.

### Informed Consent Process

The informed consent issues included the following: (1) informing subjects about their demographic and psychologic characteristics, including diagnosis; (2) exercise intervention recommendations; (3) the risks and benefits of exercise intervention; (4) the financial costs of the exercise intervention; (5) alternative services or interventions should a METH addict refuse the recommended form of care; and (6) freedom to choose or refuse exercise intervention. Written informed consent was obtained from all subjects prior to inclusion in the study. The informed consent process changed as subjects moved to different exercise training patterns. During exercise intervention, intervention providers talked with METH addicts at each juncture in exercise training when the focus or modality of exercise intervention was likely to change.

### Exercise Intervention

The METH-dependent individuals suffering from depression were further divided into two groups (depressive controls group and depressive exercise group). The exercise intervention consisted of a progressive aerobic exercise, resistance exercise training, and balance exercise program, and was conducted 5 days a week during the 12-week trial (totaling 60 sessions). Exercise sessions, which were 70 min long, were structured as follows: 5-min warm-up, 30 min of aerobic activity on a treadmill, 15 min of weight training for the major muscle groups (arms, chest, back, and legs), 10 min of BUSO ball training, and a 10-min cool down with stretching. Heart rate was continuously monitored for each participant throughout their training session using a heart rate monitor (Polar^TM^RS400, Polar Inc, Lake Success, NY, USA). The objective was to accumulate 30 min of continuous aerobic exercise in the prescribed heart rate zone. During the first 2 weeks, treadmill speed was adjusted to the target intensity between 57% HRmax to 64% HRmax; and from the third week to the twelfth week, treadmill speed was adjusted to the target intensity between 70% HRmax to 80% HRmax. Two experienced exercise trainers directly supervised all exercise sessions.

### Enzyme-Linked Immunosorbent assay

Venous blood samples were obtained from all subjects in 4-ml tubes with EDTA between 08:00 and 10:00 a.m. before breakfast and were immediately centrifuged at 3,000 rpm for 10 min. Then, plasma aliquots were stored in a freezer at −80°C until further use. Plasma BDNF, NGF, NT-3, NT-4, and proBDNF levels were measured using enzyme-linked immunosorbent assay kits (Shanghai Enzyme-linked Biotechnology Company Ltd., China).

### Purification of Peripheral Blood Mononuclear Cells

Approximately 10 ml of whole blood was collected in EDTA tubes by venous blood draw from METH-dependent depressive individuals. Peripheral blood mononuclear cells were purified by centrifugation on Histopaque-1077 (Sigma Aldrich, St. Louis, MO, USA). Briefly, peripheral blood was diluted with phosphate buffered saline, carefully layered onto Histopaque-1077, and centrifuged at 400× *g* for 30 min at room temperature. The mononuclear cell layer was then transferred to a new 15-ml conical tube and mixed with PBS to a total of 15 ml. The sample was centrifuged at 300× *g* at room temperature for 10 min with brake-on. The supernatant was then removed and discarded, and the Peripheral blood mononuclear cell (PBMC) was gently washed one more time with PBS. Consequently, cell pellet was transferred into a Nunc (Sigma-Aldrich Co., LLC) cryovial. The cryovials were directly stored at −80°C freezer overnight. The frozen cryovials were transferred and placed in liquid nitrogen.

### RNA Isolation and Quantitative Real-Time PCR

The Trizol reagent (Life Technologies, Gaithersburg, MD, USA) was used to extract the total RNA in peripheral blood mononuclear cells of 10 METH addicts with depression before and after exercise according to the manufacturer’s instructions. The concentrations and purity of RNA were measured at the optical density of 260 and 280 nm. RNA concentrations were determined with a Nano instrument (B-500 BIOPHOTOMETER, Shanghai, METASH); 3 μg total RNA was used for each reverse transcription reaction using PrimeScript real-time polymerase chain reaction (RT-PCR) kit (Takara, Japan). The reversed cDNA was served as a template and was then exposed to quantitative RT-PCR (qRT-PCR) detection using the Quanti Tect SYBR Green PCR kit (Applied Biosystems, Foster City, CA, USA) on a StepOnePlus system (Applied Biosystems). The sequences of the PCR primer pairs of TrkA, TrkB-FL, TrkB-T1, TrkC, and P75NTR are shown in Table 1. Human β-actin was amplified as an internal control for sample normalization, which has been used as a reference gene in human peripheral blood mononuclear cells in several studies (Chen et al., 1999; Ma et al., 2013; Owczarz et al., 2017). The stability of β-actin expression has been identified in peripheral blood mononuclear cells (Facci et al., 2011); previously, we also confirmed its expression stability in samples collected in this study before and after exercise by testing the CT values of β-actin in pre-experiment. PCR assays were run in triplicate for each sample. Amplifications of the PCR products were quantified by the number of cycles, and the results were analyzed using the comparative cycle threshold (Ct) method (2^−ΔCt^). The quantities of target gene expression are presented relative to the expression of the reference gene (β-actin) as individual data points using 2^−ΔCt^, where ΔCt = (Ct, _Target gene_ – Ct, _β-actin_)_._The fold change in target gene expression between the post-exercise and pre-exercise was 2^−ΔCt^, _post-exercise_/2^−ΔCt^, _pre-exercise_ (Livak and Schmittgen, 2001; Schmittgen and Livak, 2008).

**Table 1:** Degenerate primers used for quantitative real-time polymerase chain reaction (RT-qPCR).

Genes	Forward primer sequence (5′–3′)	Reverse primer sequence (5′–3′)
TrkA	TGTTGGCAGCAAGCTACATC	CGAAACGGAGACCACTCTTC
TrKB-FL	GACTACTACAGGGTCGGTGG	TTATTTGACAGCTGGTACCA
TrKB-T1	CTGTGGTGGGATTTTGCCTT	TCAACCAACAAGCACCACAG
TrkC	ACACCGGACTTCAAAAGCTG	GTGTGGTGAGCCGGTTACTT
p75NTR	GAGGCACCTCCAGAACAAGA	GCTGTTCCACCTCTTGAAGG
β-actin	CCTGGCACCCAGCACAAT	GGGCCGGACTCGTCATAC

### Statistical Analysis

Mann–Whitney U test was used to analyze differences in demographic variables between nondepression controls and depression individuals. Independent sample *t*-tests were used to analyze the different BDNF, NGF, NT3, NT4, and proBDNF plasma levels between subjects with depression and without depression after METH abstinence. Pearson correlation and Bonferroni correction for multiple testing were used to examine the relationships between SDS, SAS, or use of years and plasma levels of BDNF, NGF, NT3, NT4, and pro-BDNF, respectively. Spearman’ rank correlation and Bonferroni correction for multiple testing were applied to analyze the relationships between SDS or SAS and TrkA mRNA, TrkB-FL mRNA, TrkB-T1 mRNA, TrkC mRNA, and P75NTR mRNA in peripheral blood mononuclear cells. Analysis of covariance (ANCOVA) was used to examine group differences of SDS and SAS, and plasma levels of BDNF, NGF, NT3, NT4, and pro-BDNF from pre-exercise to post-exercise using pre-exercise values as the covariate. To estimate the practical relevance of the ANCOVA between group effects, effect sizes (eta squared, *η*^2^) were additionally calculated; the effects were defined as small (*η*^2^ = 0.01), medium (*η*^2^ = 0.06), and large (*η*^2^ = 0.14) effects according to the Cohen approach (Cohen, 1988). Paired sample *t*-test was applied to analyze the differences between pre- and post-exercise values for the exercise group. Cohen’s *d* was calculated and used for standardized mean differences for pairwise comparisons, where effect sizes were defined as small (*d* = 0.2), medium (*d* = 0.5), and large (*d* = 0.8) based on benchmarks suggested by Cohen (1988). For nonparametric test, Wilcoxon signed ranks testing was selected to determine the statistically significant differences in neurotrophin receptors of the mRNA expression between pre- and post-exercise values for the exercise intervention group. We generally considered a level of 0.05 as significant. Bonferroni test was used to correct for multiple testing. A *P*-value of < 0.01 (0.05 divided by 5, the total number of neurotrophins studied) was considered statistically significant. Descriptive data were reported as mean ± SD. All statistical analyses were performed using SPSS version 16.

## Results

### Demographic and Psychological Characteristics of METH Addicts

Seventy-two METH addicts were recruited. The demographic and psychological data of METH addicts are summarized in Table 2. The SDS and SAS scores were significantly higher in METH addicts with depression compared to METH addicts without depression (*z* = −6.960, *p* = 0.000; *z* = −6.029, *p* = 0.000). The length of use in METH addicts with depression was significantly longer than that in METH addicts without depression (*z* = −1.982, *p* = 0.047). Furthermore, there were no significant differences in age, BMI, WHR, years of education, and length of abstinence between the METH-dependent individuals with and without depression (*z* = −0.260, *p* = 0.795; *z* = −0.101, *p* = 0.920; *z* = −0.526, *p* = 0.599, *z* = −1.785, *p* = 0.074, and *z* = −0.805, *p* = 0.421).

**Table 2:** Characteristics of methamphetamine (METH) addicts with and without depression.

	Subjects		Mann–Whitney U (z)	Sig. (2-tailed)
	Non-depression (*n* = 25)	Depression (*n* = 47)		
Age (years)	31.320 (4.634)	31.521 (4.140)	553.500 (−0.260)	0.795
BMI (kg/m^2^)	24.521 (3.140)	24.408 (3.174)	579.000 (−0.101)	0.920
WHR	0.875 (0.068)	0.884 (0.051)	543.000 (−0.526)	0.599
Education (years)	9.520 (2.275)	8.617 (1.905)	453.500 (−1.785)	0.074
Length of use (years)	4.672 (2.499)	7.646 (5.318)	420.000 (−1.982)	0.047
Length of abstinence (months)	15.360 (4.100)	16.200 (3.603)	435.000 (−0.805)	0.421
SDS	39.400 (6.664)	61.510 (4.422)	0.000 (−6.960)	0.000
SAS	43.400 (10.428)	60.680 (6.695)	78.500 (−6.029)	0.000

*BMI, body mass index. The BMI is defined as the body mass divided by the square of the body height, and is universally expressed in units of kg/m^2^, resulting from mass in kilograms and height in meters. WHR, waist-to-hip ratio; SAS, Self-Rating Anxiety Scale; SDS, Self-Rating Depression Scale. Data are shown as mean (±SD)*.

### Neurotrophins Plasma Levels in METH Addicts With Depression and Without Depression

Table 3 shows the neurotrophins plasma levels in the two METH user groups (depression and nondepression). There was a clear baseline difference of plasma BDNF levels, NGF levels, NT-3 levels, and NT-4 levels between the depression group and the nondepression group; however, the pro-BDNF plasma levels were not significantly different between these two groups (*p* = 0.830, *d* = −0.05). In addition, the mean BDNF, NGF, NT-3, and NT-4 plasma levels of METH-dependent individuals were significantly higher at baseline in depressed individuals compared to the nondepressed individuals (*p* = 0.011, *d* = 0.65; *p* = 0.021, *d* = 0.59; *p* = 0.004, *d* = 0.76; *p* = 0.003, *d* = 0.78, respectively).

**Table 3:** Plasma neurotrophins levels in METH addicts with depression and without depression.

	Non-depression group (*n* = 25)	Depression group (*n* = 47)	*p*-value	Cohen’s *d* (d)
BDNF^a^	9.85 (1.65)	8.74 (1.78)	0.011	0.65
proBDNF^a^	3.05 (0.54)	3.08 (0.59)	0.830	−0.05
NGF^a^	125.83 (11.62)	116.42 (18.00)	0.021	0.59
NT-3^b^	174.99 (39.21)	146.48 (37.30)	0.004	0.76
NT-4^b^	198.69 (33.15)	172.46 (34.32)	0.003	0.78

*^a^unit: ng/ml; ^b^unit: pg/ml. Data are shown as mean (±SD)*.

**Figure 1:**
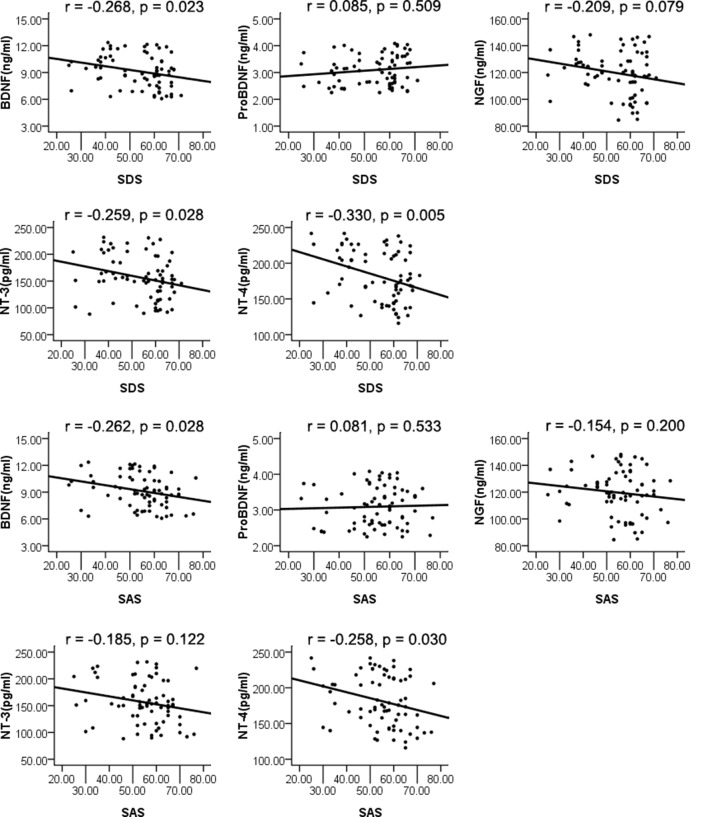
Pearson’s correlation analysis of plasma levels of neurotrophins and Self-rating Depression Scale (SDS), Self-rating Anxiety Scale (SAS).

The whole sample was analyzed using Pearson’s correlation analysis. The results are displayed in Figure 1. NT-4 plasma levels were correlated with depression (*r* = −0.330, *p* = 0.005) as measured by the SDS, which remained significant after Bonferroni correction. Plasma levels of BDNF and NT-3 were significantly correlated with depression (*r* = −0.268, *p* = 0.023; *r* = −0.259, *p* = 0.028); however, they did not reach significance after Bonferroni correction. The NGF and proBDNF plasma levels were not associated with depression (*r* = −0.209, *p* = 0.079; *r* = 0.085, *p* = 0.509). Moreover, plasma levels of BDNF and NT-4 were correlated with anxiety (*r* = −0.262, *p* = 0.028; *r* = −0.258, *p* = 0.030) as measured by the SAS; nevertheless, the difference was no longer significant after Bonferroni correction. Additionally, the NGF, NT-3, and proBDNF plasma levels were not associated with anxiety (*r* = −0.154, *p* = 0.200; *r* = −0.185, *p* = 0.122; *r* = 0.081, *p* = 0.533).

**Figure 2:**
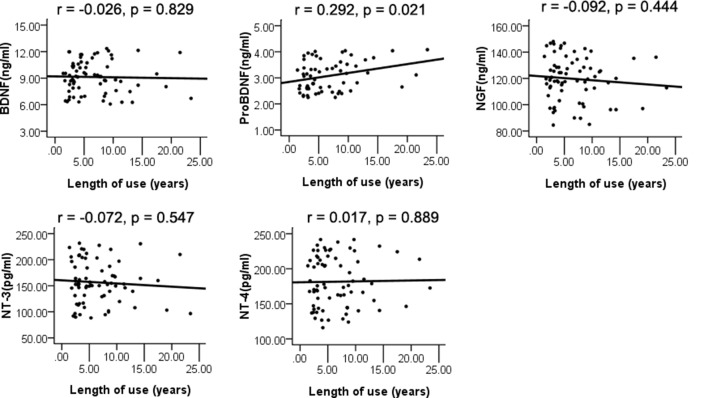
Pearson’s correlation analysis of plasma levels of neurotrophins and length of use.

The whole sample was analyzed using Pearson’s correlation analysis. The results are displayed in Figure 2. The plasma level of proBDNF was significantly correlated with the length of METH use (*r* = 0.292, *p* = 0.021), but did not pass the significance threshold for multiple hypothesis testing using the Bonferroni correction. The BDNF, NGF, NT-3, and NT-4 plasma levels were not associated with the length of METH use (*r* = −0.026, *p* = 0.829; *r* = −0.092, *p* = 0.444; *r* = −0.072, *p* = 0.547; *r* = 0.017, *p* = 0.889).

### Neurotrophins Plasma Levels in METH Addicts With Depression After Exercise Intervention

To test the changes of neurotrophins plasma levels in depressive METH addicts after exercise intervention, 47 depressive METH addicts were divided into two groups (depression control group and depression exercise group). There were no statistically significant differences between these two groups including SDS score and SAS score, and BDNF, NGF, NT-3, NT-4, and proBDNF plasma levels.

Furthermore, the ANCOVA revealed significant differences between the depression control group and the depression exercise group in BDNF, NT-3, and NT-4 plasma levels using pre-exercise values as the covariate (Tables s 4, 5). The exercise intervention revealed a significant decrease in BDNF, NT-3, and NT-4 plasma levels with effect sizes of *η*^2^ = 0.246, *η*^2^ = 0.175, *η*^2^ = 0.320 (all *p* = 0.000; see Table 5).

**Table 4:** Plasma neurotrophins levels in METH addicts among depressive control group and depressive exercise group.

Variables	Depressive control group (*n* = 20)		Depressive exercise group (*n* = 27)	
	Pre-test	Post-test	Pre-test	Post-test
BDNF^a^	8.98 (1.91)	10.27 (1.67)	8.56 (1.69)	7.58 (2.28)
proBDNF^a^	3.26 (0.60)	2.94 (0.56)	3.91 (0.56)	4.00 (0.51)
NGF^a^	120.28 (20.00)	107.36 (17.01)	113.56 (16.15)	113.00 (20.74)
NT-3^b^	150.20 (37.30)	183.04 (31.96)	143.72 (37.76)	147.59 (35.44)
NT-4^b^	176.86 (35.67)	230.82 (39.03)	169.20 (33.59)	171.74 (37.73)

*^a^unit: ng/ml; ^b^unit: pg/ml. Data are shown as mean (±SD)*.

**Table 5:** Analysis of covariance for the efficacy of exercise intervention.

Variables source		Type III sum of squares	Observed power	*F*	Sig	Eta Square (*η*^2^)
BDNF	Pre-test	110.273	1.000	61.653	0.000	0.440
	Between groups	61.719	1.000	34.507	0.000	0.246
	Error	78.698	-	-	-	0.314
	Total	250.690	-	-	-	-
proBDNF	Pre-test	0.042	0.066	0.145	0.706	0.004
	Between groups	0.010	0.054	0.035	0.852	0.001
	Error	10.154	-	-	-	0.995
	Total	10.206	-	-	-	-
NGF	Pre-test	7,980.478	1.000	40.324	0.478	0.445
	Between groups	1,255.641	0.693	6.345	0.126	0.070
	Error	8,707.977	-	-	-	0.485
	Total	17,944.096	-	-	-	-
NT-3	Pre-test	28,541.988	1.000	53.380	0.000	0.452
	Between groups	11,032.575	0.993	20.633	0.000	0.175
	Error	23,526.477	-	-	-	0.373
	Total	63,101.040	-	-	-	-
NT-4	Pre-test	41,618.525	1.000	75.203	0.000	0.429
	Between groups	31,065.059	1.000	56.133	0.000	0.320
	Error	24,350.242	-	-	-	0.251
	Total	97,033.826	-	-	-	-

### Neurotrophin Receptors in Peripheral Blood Mononuclear Cells in METH Addicts With Depression After Exercise Intervention

The top 10 METH addicts whose SDS score decreased after exercise intervention by purposive sampling were selected to examine the change of neurotrophin receptor mRNA in peripheral blood mononuclear cells, and the relationship between neurotrophin receptor mRNA levels and depression after exercise training intervention. As shown in Table 6, the fold change in TrkB-FL and TrkB-T1 mRNA expression in peripheral blood mononuclear cells between the post-exercise and pre-exercise demonstrated a remarkable decrease (fold change = −11.056 and −39.055), while the P75NTR mRNA expression showed a mild decline (fold change = −2.089). Moreover, Wilcoxon signed ranks testing indicated a significant difference for TrkB-FL mRNA levels (*z* = −2.293, *p* = 0.022) and TrkB-T1 mRNA levels (*z* = −2.497, *p* = 0.013) in peripheral blood mononuclear cells between post-exercise and pre-exercise. A slightly significant difference for P75NTR mRNA expression (*z* = −1.886, *p* = 0.059) was detected between pre- and post-exercise. There were no significant differences between TrkA and TrkC mRNA expression (*z* = −1.274, *p* = 0.203 and *z* = −0.968, *p* = 0.333) pre- and post-exercise (Table 7).

**Table 6:** Neurotrophin receptors mRNA in peripheral blood mononuclear cells in depressive METH addicts before and after exercise (*n* = 10).

mRNA	Pre-exercise	Post-exercise	Fold change
TrKA	8.139E-5 (6.402E-5)	1.013E-4 (6.557E-5)	1.245
TrKB-FL	4.867E-3 (6.431E-3)	4.402E-4 (1.187E-3)	−11.056
TrKB-T1	3.780E-4 (7.457E-4)	9.679E-6 (6.114E-6)	−39.055
TrKC	8.429E-5 (6.201E-5)	1.013E-4 (6.557E-5)	1.202
P75NRT	4.420E-5 (4.255E-5)	2.115E-5 (1.217E-5)	−2.089

*Neurotrophin receptors mRNA are shown as relative value. Data are shown as mean (±SD)*.

**Table 7:** Wilcoxon Signed Ranks Test of neurotrophin receptors mRNA in peripheral blood mononuclear cells in depressive METH addicts before and after exercise (*n* = 10).

mRNA	Negative ranks (N)	Positive ranks (N)	z	Sig. (2-tailed)
TrKA_post-exercise_-TrKA_pre-exercise_	3	7	−1.274^a^	0.203
TrKBFL_post-exercise_-TrKBFL_pre-exercise_	9	1	−2.293^a^	0.022
TrkBT1_post-exercise_-TrkBT1_pre-exercise_	8	2	−2.497^a^	0.013
TrKC_post-exercise_-TrKC_pre-exercise_	3	7	−0.968^a^	0.333
P75NRT_post-exercise_-P75NRT_pre-exercise_	7	3	−1.886^a^	0.059

*^a^Based on negative ranks*.

**Figure 3:**
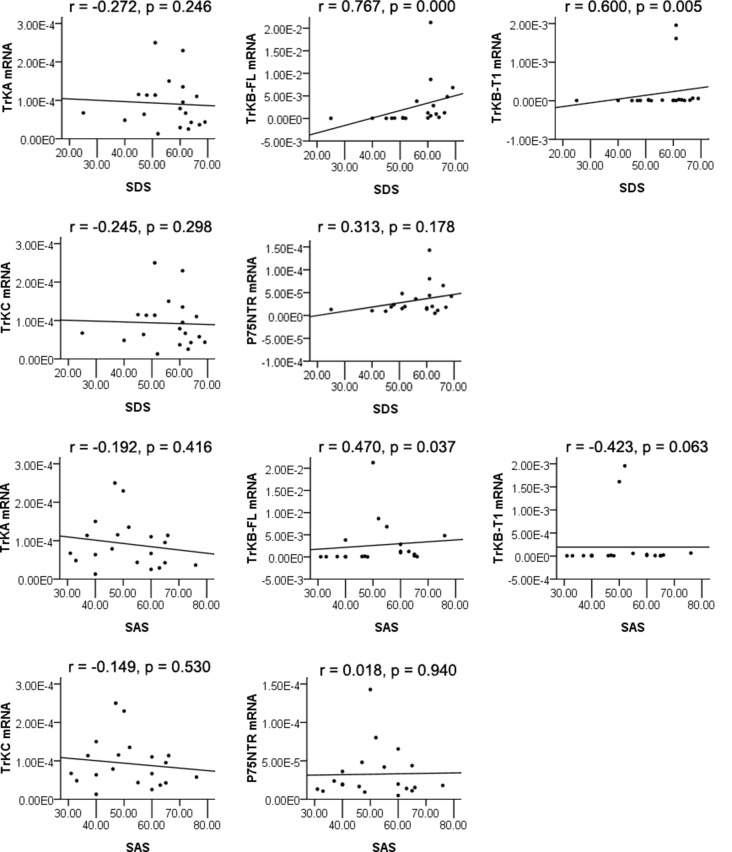
Spearman’s rank correlation analysis of neurotrophin receptors mRNA in PBMCs and SDS, SAS.

We found a significantly positive correlation between TrkB-FL mRNA levels and depression (*r* = 0.767, *p* = 0.000) as measured by the SDS, as well as TrkB-T1 mRNA levels and depression (*r* = 0.600, *p* = 0.005). Statistical significance was reached after correcting for multiple testing (*p* < 0.01 after Bonferroni correction). The changes of TrkB-FL mRNA and TrkB-T1 mRNA in peripheral blood mononuclear cells revealed to be a good predictor of depression after exercise intervention for METH addicts, while correlations were not found between SDS and TrkA mRNA, TrkC mRNA, and P75NTR mRNA (*r* = −0.272, *p* = 0.246; *r* = −0.245, *p* = 0.298; *r* = 0.313, *p* = 0.178, respectively) among depressive exercise training individuals (Figure 3). Besides, TrkB-FL mRNA levels were positively correlated with anxiety (*r* = 0.470, *p* = 0.037; the difference was no longer significant after Bonferroni correction) as measured by the SAS after exercise intervention for METH addicts, while TrkA mRNA, TrkB-T1 mRNA, TrkC mRNA, and P75NTR mRNA levels were not associated with anxiety (*r* = −0.192, *p* = 0.416; *r* = −0.423, *p* = 0.063; *r* = −0.149, *p* = 0.530; *r* = 0.018, *p* = 0.940; see Figure 3).

## Discussion

After METH withdrawal, some mood-related symptoms may resolve or decrease during the first few weeks (Iudicello et al., 2010; Zorick et al., 2010), while others may extend over a year and sometimes even longer (Iudicello et al., 2010). Nakama et al. (2008) and Shen et al. (2012) found that participants who experience depression and anxiety, which last longer than 1 year, can negatively affect the rehabilitation from METH abstinence, and eventually lead to relapse. In this study, we further examined the relationship between neurotrophins and depression among METH users that suffered from depression for more than 1 year compared to those who did not. In addition, the effects of exercise intervention on neurotrophins and their receptors for depression and anxiety were also examined. Besides the length of use in years that was significantly longer in depressive METH addicts compared to nondepressive METH addicts (*z* = −1.982, *P* = 0.047), there were no statistically significant differences among other demographic characteristics including age, BMI, WHR, years of education, and length of abstinence (as shown in Table 2).

Although the role of neurotrophins, including BDNF, NGF, NT-3, NT-4, and glial cell line-derived neurotrophic factor (GDNF) in depressive-like behavior has been well studied (Lin and Tseng, 2015; Loch et al., 2015; Wiener et al., 2015; Björkholm and Monteggia, 2016; Wysokiński, 2016), studies investigating neurotrophins levels among METH addicts are still lacking. Ren et al. (2016) have found that METH users have significantly higher serum BDNF levels compared to healthy controls at baseline during early withdrawal (1–7 days long abstinence); in addition, they found a time-dependent decrease in serum BDNF in METH addicts during the first month of withdrawal. Moreover, Chen et al. (2015) found significantly lower serum BDNF levels in METH addicts during early withdrawal (abstinent for ≤21 days) compared to healthy controls. As shown in an animal model, repeated METH administration to mice caused a long-lasting depression-like behavior. Western blot analysis showed that BDNF levels in the nucleus accumbens of METH-treated depressive mice were significantly higher than those of control mice, whereas BDNF levels in other regions, including the prefrontal cortex and hippocampus, were not altered (Ren et al., 2015). In contrast, chronic METH abuse has shown to increase plasma BDNF concentrations after 30 or more days of METH abstinence (Kim et al., 2005). All these data suggest the BDNF levels in METH abusers are regulated by complex mechanisms. Our study showed that overall BDNF, NGF, NT-3, and NT-4 levels are higher in METH users with depression compared to METH addicts without depression (*d* = 0.65, 0.59, 0.76, 0.78, respectively, as shown in Table 3). Following Cohen (1988), we interpreted estimated Cohen’s *d* values as follows: 0.2 small effect size, 0.5 medium effect size, and 0.8 large effect size. Therefore, there were medium to large sized effects for the differences of BDNF, NGF, NT-3, and NT-4 between METH users with depression and without depression, whereas no difference was found in pro-BDNF levels (*p* = 0.830, *d* = −0.05). Moreover, a correlation analysis revealed a negative relation between depression and NT-4 (*r* = −0.330, *p* = 0.005), and a weak negative relation between anxiety and BDNF or NT-4 (*r* = −0.268, *p* = 0.023 or *r* = −0.259, *p* = 0.028) in METH addicts. Based on the differences in the years of METH use among depressive abusers and nondepressive abusers in this study, we explored the correlation between neurotrophins and the length of METH use. Consistent with the study results reported by Ren et al. (2016), we found that the plasma levels of BDNF were not correlated with the years of METH use. In addition, we also found that the NGF, NT-3, and NT-4 plasma levels were not associated with the years of use, while plasma levels of proBDNF were correlated with the length of use (*r* = 0.292, *p* = 0.021); however, these correlations did not pass the significance threshold adjusted by the Bonferroni correction. Because the ratio of BDNF to proBDNF has a role in depressive-like behaviors (Qiao et al., 2017; Zhao et al., 2017), and considering the putative opposing functions of BDNF and proBDNF, it would be clinically and scientifically useful to further investigate the conversion of proBDNF to BDNF. Our results, which are consistent with previous studies, suggest that BDNF and NT-4 are correlated with psychiatric symptoms such as depression and anxiety.

Clinical and preclinical testing demonstrated that exercise training affects METH consumption and craving in humans and animals (Mooney et al., 2014; Aarde et al., 2015; Haglund et al., 2015; Rawson et al., 2015; Damghani et al., 2016; Wang et al., 2016); yet, its exact mechanism of action still remains unclear. In this study, we applied a 12-week structured program of progressive aerobic and resistance exercise training together with balanced exercise in METH-dependent individuals with and without depression. We discovered that this particular exercise training produces positive effects by reducing mood-related symptoms of depression and anxiety by decreasing the score of SDS and SAS, and decreasing the levels of plasma BDNF, NT-3, and NT-4 compared with depressive individuals without exercise (*η*^2^ = 0.246, 0.175, 0.320, respectively, as illustrated in Table 5). Moreover, we found that the exercise intervention influenced changes of plasma BDNF, NT-3, and NT-4, which were classified according to Cohen (1988) into small (*η*^2^ = 0.01), medium (*η*^2^ = 0.06), and large (*η*^2^ = 0.14) effects, while the levels of plasma pro-BDNF and NGF remained unchanged in depressive addicts with exercise. These data provide new evidence that structural regular exercise can decrease the BDNF, NT-3, and NT-4 plasma levels that are correlated with the alleviation of depression and anxiety after METH withdrawal.

Next, we investigated the expression of the high affinity receptors TrkA, TrkB, and TrkC as well as the low affinity pan neurotrophin receptor p75NTR in human peripheral blood mononuclear cells. Briefly, we found a decreased expression of *full-length* form and *truncated* form of TrkB mRNA and a slightly decreased expression of p75NTR mRNA in PBMC in METH abstinence individuals who exercised, while the expression of TrkA and TrkC remained unchanged. This finding is consistent with Ren et al. (2015), who found that blocking BDNF–TrkB signaling in the nucleus accumbens shell of mice represents a potential therapeutic approach for behavioral abnormalities after repeated METH exposure.

With reference to the TrkB receptor, in addition to the active *full-length* form of the receptor (TrkB-FL), a *truncated* form (TrkB-T1) was found to lack kinase activity and inhibit the function of TrkB-FL by competing in binding to BDNF (Carim-Todd et al., 2009). TrkB-FL signaling is regulated by neuronal survival and differentiation via PLCy, PI3K, and Erk/MAPK pathways (Fenner, 2012). TrkB-T1, on the other hand, interacts with a Rho GDP dissociation inhibitor (GDI); the binding of BDNF to TrkB-T1 leads to the release of the Rho-GDI and, in turn, in the inhibition of Rho (Ohira et al., 2005). Analysis of mice lacking selectively truncated form of TrkB revealed a decrease in dendritic complexity specifically within the amygdala. This defect in neuronal morphology was associated with an increase in anxiety (Carim-Todd et al., 2009; Deinhardt and Chao, 2014). An excess of the TrkB-T1 isoform can lead to neuronal death (Vidaurre et al., 2012), whereas the elevated levels of truncated isoforms have been found in the prefrontal cortex of individuals with schizophrenia (Wong et al., 2013). Repeated administration of METH leads to a marked increase in BDNF–TrkB signaling in the nucleus accumbens shell, eventually resulting in the long-lasting depression-like behavior that was observed in mice after METH withdrawal (Berton et al., 2006; Zhang et al., 2014; Wook Koo et al., 2016; Qiao et al., 2017). Considering that the currently available human and animal results have proposed that the enhanced BDNF-TrkB signaling in lymphocytes parallels BDNF-TrkB cortical activity (Wang et al., 2011), our findings suggested that a marked increase in BDNF within plasma by METH exposure contributes to long-lasting behavioral abnormalities (depression and anxiety). In the present study, we found a decrease in TrkB-FL mRNA and TrkB-T1 mRNA of 11.056- and 39.055-fold, respectively in METH-dependent individuals who were exercising compared with controls who did not. The effect of the functioning BDNF receptors (TrkB FL/TrkB T1 ratio, FL/T1 thereafter) was analyzed in relation to antipsychotics and the clinical status of patients, indicating that the presence of a higher FL/T1 ratio in patients was associated with a better clinical response to antipsychotics (Martinez-Cengotitabengoa et al., 2016). Through TrkB activation, BDNF promotes maturation of the immune system as well as development, maintenance, and survival of lymphocytes (Schuhmann et al., 2005). The link between the immune system and the brain does not come as a surprise, as numerous studies have shown that the two systems can interact bidirectionally (Derecki et al., 2010; Yirmiya and Goshen, 2011). In this study, the FL/T1 ratio increased 3.532-fold (39.055/11.056) in depressive METH-abstinence exercised subjects whose SDS scores decreased due to exercise intervention when compared with pre-exercise condition. Correlation analysis in this study revealed a significant and positive relation between TrkB-FL mRNA levels and depression (*r* = 0.767, *p* = 0.000), as well as TrkB-T1 mRNA levels and depression (*r* = 0.600, *p* = 0.005). These results revealed that 12-week exercise training could be attributed to improved depressive symptoms of METH withdrawal, which was correlated with the BDNF-TrkB pathway in peripheral blood mononuclear cells.

## Conclusions

This study provides novel evidence that the increase in neurotrophins plasma levels, including BDNF and NT-4, is correlated with the occurrence of depression and anxiety during long-term recovery after METH abstinence. On the other hand, 12-week structural regular exercise training integrated with aerobic, resistance, and balanced exercise can decrease the BDNF, NT-3, and NT-4 plasma levels that are correlated with the alleviation of depression and anxiety after METH withdrawal. In addition, we found that exercise intervention decreased the expression of TrkB mRNA and increased the FL/T1 ratio of TrkB mRNA in peripheral blood mononuclear cells of depressive METH abstinence individuals; a correlation analysis uncovered a significant and positive relation between TrkB-FL mRNA levels or TrkB-T1 mRNA levels and depression. We speculated that exercise intervention could be attributed to improved depressive symptoms of METH withdrawals, which was correlated with the BDNF-TrkB pathway in peripheral blood mononuclear cells. Yet, the expression of TrkB on peripheral blood mononuclear cells should be further investigated not only as a marker but also as the role of inflammation in METH dependence with depression before and after exercise intervention.

## Data Availability Statement

All datasets generated for this study are included in the article.

## Ethics Statement

The studies involving human participants were reviewed and approved by The Institutional Review Board at the Hunan Normal University and who also approved the study protocol. The patients/participants provided their written informed consent to participate in this study.

## Author Contributions

JY, LZ, XL, and JZ conceived and designed the experiments. JT, CL, WH, and YL screened experimental subjects, signed the informed consent process, and carried out exercise intervention. QL, JL, and DC performed blood collection and separation, and carried out quantitative real-time PCR. JY and LZ analyzed the data. JY and JT wrote the manuscript. All authors have read and approved the final manuscript.

## Conflict of Interest

The authors declare that the research was conducted in the absence of any commercial or financial relationships that could be construed as a potential conflict of interest.
